# Supervised neuromuscular exercise prior to hip and knee replacement: 12-month clinical effect and cost-utility analysis alongside a randomised controlled trial

**DOI:** 10.1186/s12891-016-1369-0

**Published:** 2017-01-06

**Authors:** Linda Fernandes, Ewa M. Roos, Søren Overgaard, Allan Villadsen, Rikke Søgaard

**Affiliations:** 1 Department of Orthopaedic Surgery and Traumatology, Odense University Hospital, Odense, Denmark; 2 Department of Rehabilitation, Odense University Hospital, Sdr. Boulevard 29, 5000 Odense C, Denmark; 3 Research Unit for Musculoskeletal Function and Physiotherapy, Institute of Sports Science and Clinical Biomechanics, University of Southern Denmark, Odense, Denmark; 4 Department of Clinical Research, University of Southern Denmark, Odense, Denmark; 5 Department of Public Health, Aarhus University, Aarhus, Denmark; 6 Department of Clinical Medicine, Aarhus University, Aarhus, Denmark

**Keywords:** Osteoarthritis, Exercise, Cost-benefit analysis, Arthroplasty, Replacement

## Abstract

**Background:**

There are indications of beneficial short-term effect of pre-operative exercise in reducing pain and improving activity of daily living after total hip replacement (THR) and total knee replacement (TKR) surgery. Though, information from studies conducting longer follow-ups and economic evaluations of exercise prior to THR and TKR is needed. The aim of the study was to analyse 12-month clinical effect and cost-utility of supervised neuromuscular exercise prior to THR and TKR surgery.

**Methods:**

The study was conducted alongside a randomised controlled trial including 165 patients scheduled for standard THR or TKR at a hospital located in a rural area of Denmark. The patients were randomised to replacement surgery with or without an 8-week preoperative supervised neuromuscular exercise program (Clinical Trials registration no.: NCT01003756). Clinical effect was measured with Hip disability and Osteoarthritis Outcome Score (HOOS) and Knee injury and Osteoarthritis Outcome Score (KOOS). Quality adjusted life years (QALYs) were based on EQ-5D-3L and Danish preference weights. Resource use was extracted from national registries and valued using standard tariffs (2012-EUR). Incremental net benefit was analysed to estimate the probability for the intervention being cost effective for a range of threshold values. A health care sector perspective was applied.

**Results:**

HOOS/KOOS quality of life [8.25 (95% CI, 0.42 to 16.10)] and QALYs [0.04 (95% CI, 0.01 to 0.07)] were statistically significantly improved. Effect-sizes ranged between 0.09-0.59 for HOOS/KOOS subscales. Despite including an intervention cost of €326 per patient, there was no difference in total cost between groups [€132 (95% CI −3942 to 3679)]. At a threshold of €40,000, preoperative exercise was found to be cost effective at 84% probability.

**Conclusion:**

Preoperative supervised neuromuscular exercise for 8 weeks was found to be cost-effective in patients scheduled for THR and TKR surgery at conventional thresholds for willingness to pay. One-year clinical effects were small to moderate and favoured the intervention group, but only statistically significant for quality of life measures.

**Trial registration:**

ClinicalTrials.gov (NCT01003756) October 28, 2009.

## Background

Total hip and knee replacement (THR and TKR) surgery are recognized treatments for pain relief in patients with severe symptoms from hip or knee osteoarthritis (OA) [1]. Nevertheless, one year after surgery up to 50% of patients undergoing THR and TKR may not experience clinically important improvements in pain and activities of daily living (ADL) [2, 3]. Supervised exercise has shown to be effective treatment for reducing pain and improving ADL in patients with OA [4–9]. It seems exercise at later stages of the disease, and prior to joint replacement surgery, also has beneficial results [10, 11].

However, before a new treatment strategy such as pre-operative exercise is implemented, one key input into the decision-making process is the effect and cost-effectiveness of the strategy in question. Today, information on the post-operative effect of exercise prior to surgery is sparse and sufficiently powered studies with feasible interventions and longer follow-ups along with high-quality economic evaluations are warranted [11–15].

We previously conducted a randomised controlled trial (RCT) evaluating an 8-week supervised neuromuscular exercise prior to THR and TKR [10, 16]. The study showed overall improvements in favour of the exercise group in ADL prior to surgery and at 6 weeks postoperatively. At 3 months postoperatively the effects were diminished [16]. Although demonstrating short-term effects only, the addition of preoperative exercise may be clinically important in early mobilisation and returning to prior activities. Our aim with this study was to evaluate one-year clinical effect and cost-utility of the supervised neuromuscular exercise programme prior to THR and TKR. If supervised exercise prior to THR and TKR is shown to be cost-effective, health policy decision makers should consider changing the pre-operative care trajectory to include supervised exercise prior to THR and TKR.

## Methods

### Overview of study design and participants

**Fig. 1:**
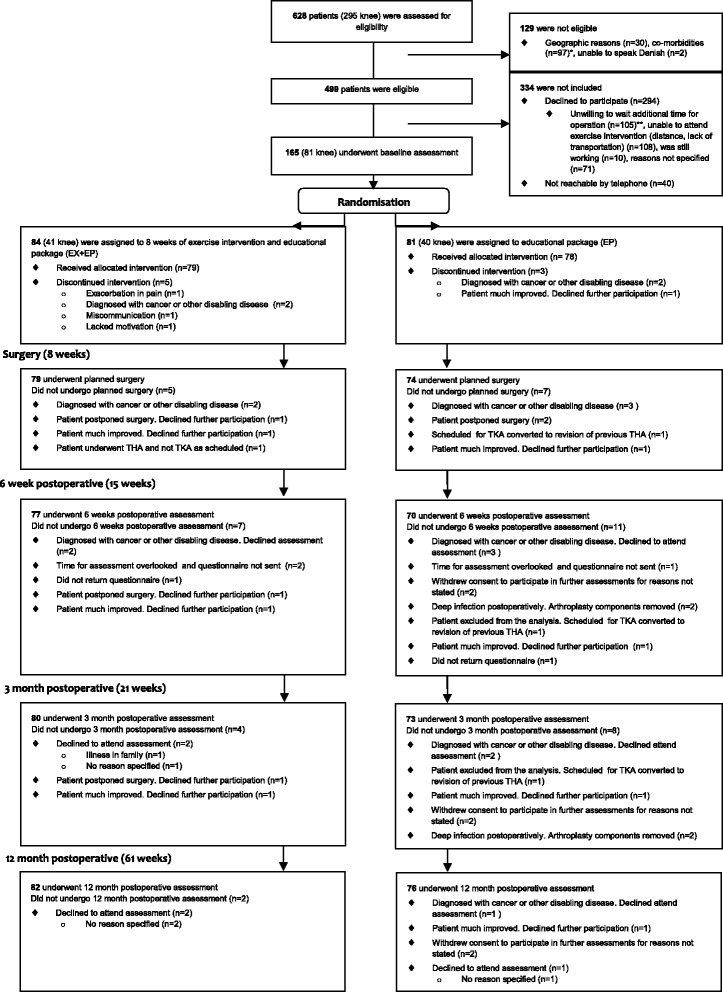
Flow diagram of patients participating in this study. * Co-morbidities (n = 97). ◆ Previous fracture in or adjacent to the joint (*n* = 13) (1 knee). ◆ Inflammatory arthritis (*n* = 11) (5 knee). ◆ Revision arthroplasty (*n* = 7) (4 knee). ◆ Previously enrolled with another joint (*n* = 9) (7 knee). ◆ Unicompartemental replacement (knee) (*n* = 27). ◆ Bilateral procedure in same session or within 3 month (*n* = 16). ◆ Necrosis of the femoral head (hip) (*n* = 6). ◆ Neurological disorders (*n* = 6), Hemiparesis (*n* = 2), Parkinsons Disorder (*n* = 2), Dementia (n = 2). ◆ Dysplasia of the femoral head (*n* = 1). ◆ Possible cancer metastasis in proximal femur (*n* = 1). ** The Danish Healthcare System has a one month treatment guarantee. Entering this study meant all patients accepting an additional wait of up to 5 weeks in comparison to the treatment guarantee. After randomization, this additional wait applied only for patients randomized to the 8 week exercise intervention. The control group was operated on when originally scheduled

165 patients were included between 4 January 2010 and 21 March 2011.[10, 16] Inclusion criteria were; ≥18 years of age and scheduled for THR or TKR due to symptomatic OA. Exclusion criteria were; scheduled for bilateral surgery, previous fractures in or adjacent to the joint, inflammatory arthritis and severe heart disease or neurologic deficits. Included patients were randomly allocated to the intervention group, i.e. supervised neuromuscular exercise and preoperative educational package (EP); or to the control group, i.e. EP alone (Fig. 1). The primary outcome was the ADL subscale of the Hip disability and Osteoarthritis Outcome Score (HOOS) and Knee injury and Osteoarthritis Outcome Score (KOOS) [17–19].

Clinical effect was measured with HOOS and KOOS at one year post-surgery. Assessments points were at baseline, 8 weeks (post-intervention), 15 weeks (6 weeks post-surgery), 21 weeks (3 months post-surgery) and 61 weeks (one year post-surgery). The economic evaluation was conducted alongside the RCT and applied the health care sector perspective, incorporating cost of health care services, including cost of the exercise program. It took form of a cost-utility analysis using Quality Adjusted Life Years (QALYs). The time horizon was 61 weeks within the start and end date of the study (4 January 2010 – 13 August 2012).

### Intervention

The neuromuscular exercise programme was supervised by a physiotherapist and focused on lower extremity muscular control and quality of movement.[10, 16, 20, 21] It consisted of three parts: warm-up, circuit programme and cool-down. The majority of the exercises were weight-bearing exercises imitating functions of daily living and the patients learned how to control hip-knee-foot alignment in each exercise. Progression of exercise level was guided by neuromuscular control and quality of the performance (determined by the physiotherapist) and with acceptable exertion (determined by the patient). The programme was delivered in groups of 6–12 patients twice weekly lasting 1 h per session at the Department of Rehabilitation at Odense University Hospital, Svendborg, in a rural part of Southern Denmark. An attendance of 12 sessions or more was considered good compliance.

The EP was standard preoperative information on the operating procedure, expected postoperative progression and a leaflet on various exercises [16]. All patients were offered the EP.

### Intervention cost

Valuation of formal care of the exercise program was based on tariff-based costs for physiotherapy in primary care (https://fysio.dk/globalassets/documents/raadgivning/overenskomster/praksisoverenskomster/takster-for-fysioterapi-oktober-2016.pdf). The fees reflect the physiotherapist’s wage, capital cost and expenses, e.g. rental costs, use of equipment, dispensable material and electricity. Implementation cost of the exercise program was not included. Costs for the 3-h patient education package were not included as this was offered to all participants in the trial. All monetary units were reported in 2012-EUR with an exchange rate of DKK 7.45 to 1 EUR.

### Health care utilisation and cost

Individual data was extracted from two national registers: The National Health Insurance Service Registry and The Danish National Patient Register [22, 23]. The former includes details about all services provided in primary care including national reimbursement fees [22]. The latter includes details about all contacts to hospitals including diagnoses, procedures and diagnosis-related-grouping casemix tariffs [23].

### Patient expenses

Valuation of patients’ time and transport for attending the exercise classes were included in a sensitivity analysis. Valuation of informal time (i.e. time spent by patients attending the exercise regimen) was based on a human capital approach, for which the value of a person’s time is reflected by wage rates (productivity loss). The wage rate was estimated by applying age- and gender matched national average gross income for year 2012 extracted from Statistics Denmark [24]. Informal time for one exercise session was set to a fixed value of 1.25 h. Patients’ expenses for travelling to and from the gym were calculated by using the national fees for travel reimbursement for 2012 (DKK 3.80/km or €0.51/km) times the distance (km) between the exercise facility and patients’ homes.

### Patient reported outcome measures

The HOOS and KOOS assesses pain, symptoms, ADL, function in sport and recreation and knee related quality of life in five separate subscales scored on a 0–100 (worst to best) scale [18, 19, 25, 26].

Utility was expressed as QALYs measured with the generic outcome measure European Quality of Life 5-Dimension 3-Level Health Outcome (EQ-5D-3L) [27]. Health state valuations from the Danish general population were adopted [28].

### Analysis

Baseline subject characteristics were summarised as number (%) or mean (SD). QALYs were produced by calculating the area under the curve of the EQ-5D-3L utility scores from baseline and all follow-ups assuming linear trend between observations. Visits and costing for primary care were categorised based on care provider. In hospital stay and costing for secondary care were categorised based on primary unit. All parameters were tested for normality and distribution. Because of skewed data all comparative analyses, including the net benefit, were based on bootstrapped standard errors. Non-parametric bootstrapping with 10,000 replications was applied [29].

Group comparisons were based on intention-to-treat analysis.[30] Since no interaction (group allocation ˣ joint involved) was seen in the original RCT,[16] the analyses did not adjust for hip or knee involvement. One-year clinical effect was expressed as the between-group mean difference [95% confidence interval (CI)] of change values (61 weeks – baseline) and effect-size (d = mean difference of change values/pooled baseline standard deviation) of the five subscale scores of the HOOS and KOOS. Analysis of linear regression was used for between-group comparisons of QALYs and costs and presented as between-group mean differences (95% CI) over the time horizon. An adjustment for baseline health utility was included in the analysis to account for baseline imbalances in the estimation of mean differential QALYs [31].

Handling of missing EQ-5D-3L utility scores was based on comparison of complete item response and two different imputation methods: last observation carried forward (LOCF), in which missing values are imputed based on existing values, and linear trend at point (LTAP), in which missing values are imputed by values based on a linear regression model using nonmissing observations in the series to fit the regression. Analysis comparing responders and non-responders was performed for QALYs. Since imputed data using the LTAP method showed the lowest mean difference estimate for QALYs (Table 2), and thereby the lowest risk of overestimating results, it was decided to be used in the analyses. A significance level of 0.05 was used.

#### Cost-utility

The cost-utility analysis adopted a health care sector perspective. We estimated the value for money of the intervention by calculating the incremental net monetary benefit using a range of hypothetical threshold values for decision-makers’ willingness-to-pay for a unit of effect [32]. The threshold values ranged from €0 to €100,000. The net benefits were presented visually in cost-effectiveness acceptability curves (CEAC). These curves illustrate the probability that the intervention is cost-effective compared to the control at various threshold values of willingness-to-pay for a QALY gain. A willingness-to-pay of €40,000 per QALY gained was used as the threshold indicating good value for money [33, 34].

#### Sensitivity analyses

Four sensitivity analyses were performed to assess robustness of results for cost-utility and presented in the CEAC.

1. Complete item response analysis leaving out patients not filling in the EQ-5D-3L one or more times during the follow-up period.
2. Per-protocol analysis including only patients who complied to exercise.
3. Not adjusting analysis for the potentially skewed baseline EQ-5D-3 L.
4. Including patients’ travel and time costs associated with attending exercise.

## Results

Overall, 92.1% of the observations of the HOOS or KOOS and the EQ-5D-3L at the five assessment points were complete. There were 122 (74%) complete item responses for QALYs. There was no significant difference between number of patients with missing QALY in the intervention (*n* = 21) and control group (*n* = 22) (*p* = 0.75). Cost data had no missing values. Except for the EQ-5D-3L, there were no differences at baseline. Baseline characteristics are presented in Table 1. Five patients did not go through THR or TKR surgery during the 61 weeks. Reasons for declining surgery were: intervention group, much improved after exercise (*n* = 1) and no reason specified (*n* = 1); and control group, cancer (*n* = 1), started to exercise on her own (*n* = 1) and anxious about surgical procedure (*n* = 1).

**Table 1:** Baseline subject characteristics

		Intervention group	Control group	
				Mean difference (95%CI)
n		84	81	
Female sex		47 (56%)	45 (56%)	
Age–years		67.9 (8.6)	66.9 (8.3)	1.08 (−1.52 to 3.67)
BMI–kg/m^2^		29.6 (4.5)	31.1 (6.1)	−1.41 (−3.05 to 0.24)
Waiting list THA		43 (51%)	41 (51%)	
Waiting list TKA		41 (49%)	40 (49%)	
EQ-5D-3L		0.63 (0.15)	0.57 (0.19)	0.06 (0.01 to 0.12)
HOOS/KOOS				
	ADL	50.5 (15.1)	45.6 (16.9)	4.9 (−0.02 to 9.83)
	Pain	46.8 (14.3)	42.7 (14.4)	4.0 (−0.39 to 8.46)
	Symptoms	49.4 (19.7)	44.6 (18.6)	4.8 (−0.98 to 10.55)
	Sport & Recreation	24.6 (17.2)	19.9 (18.2)	4.6 (−0.80 to 10.09)
	Quality of life	31.2 (12.1)	28.9 (15.9)	2.3 (−2.01 to 6.60)

Continuous variables are expressed as the mean (SD); non-continuous variables are expressed as the number of patients (%); *THA*, Total hip arthroplasy; *TKA*, Total knee arthroplasty; *EQ5D*-*3L*, European Quality of Life 5 Dimension 3 Level Health Outcome; *HOOS*, Hip disability and Osteoarthritis Outcome Score; *KOOS*, Knee injury and Osteoarthritis Outcome Score; *ADL*, function in daily living

### Patient reported outcomes

The intervention was associated with a statistically significant QALY gain of 0.04 (95% CI, 0.01 to 0.07). The QALYs showed similar results for complete item response, different imputation methods and unadjusted analyses (Table 2). Mean differences at 61 weeks favoured the exercise group for all HOOS/KOOS subscales, however only significantly so for the quality of life subscale [mean difference 8.25 points (95% CI, 0.42 to 16.10)] (Table 2). Effect-sizes for HOOS/KOOS subscales ranged from 0.09 – 0.59 (Table 2).

**Table 2:** Mean differences of QALYs and HOOS or KOOS for the 61-week follow-up period

	Intervention (*n* = 84)		Control (*n* = 81)			
	*n*	Mean (SE)	*n*	Mean (SE)	Mean difference (95% CI)	ES
QALY, LTAP^a^	84	0.66 (0.04)	81	0.61 (0.04)	0.04 (0.01 to 0.07)	
QALY, LTAP^b^	84	0.80 (0.01)	81	0.74 (0.01)	0.06 (0.02 to 0.09)	
QALY, LOCF^a^	82	0.64 (0.05)	79	0.58 (0.05)	0.05 (0.02 to 0.09)	
QALY, complete^a^	63	0.68 (0.05)	59	0.63 (0.05)	0.05 (0.02 to 0.09)	
QALY, per-protocol^a^	62	0.63 (0.04)	81	0.59 (0.05)	0.04 (0.01 to 0.07)	
HOOS/KOOS						
ADL	84	35.9 (2.1)	81	32.1 (2.4)	3.80 (−2.45 to 10.07)	0.24
Pain	84	41.2 (2.2)	81	37.1 (2.4)	4.13 (−2.33 to 10.60)	0.29
Symptoms	84	33.9 (2.7)	81	32.2 (2.6)	1.69 (−5.79 to 9.17)	0.09
Sport and Recreation	84	33.1 (3.6)	81	26.3 (2.8)	6.79 (−2.10 to 15.69)	0.39
Quality of Life	84	43.4 (2.6)	81	35.2 (3.0)	8.25 (0.42 to 16.10)	0.59

Mean (bootstrap SE) and mean differences (95% confidence interval). ES, effect-size (mean difference/pooled standard deviation)

*QALY*, quality-adjusted life-years; complete, complete item response analysis; *LTAP*, linear trend at point; *LCOF*, last observation carried forward; per-protocol, per-protocol analysis equals attending ≥12 exercise sessions. *HOOS*, Hip disability and Osteoarthritis Outcome Score; *KOOS*, Knee injury and Osteoarthritis Outcome Score; *ADL*, function in daily living

^a^Adjusted for baseline EQ-5D-3 L scores

^b^Unadjusted analysis

### Resource use

All patients in both groups attended the preoperative education package prior to surgery. In total, 144 exercise sessions were provided during the intervention period with a mean of 7.7 patients per session. On average, patients in the intervention group had attended the exercise programme 13.1 times (Table 3). Sixty-two of the 84 patients in the intervention group (74%) displayed good compliance.

**Table 3:** Health care utilization during 61 weeks

		Intervention group (*n* = 84)	Control group (*n* = 81)	
				Mean difference (95% CI)
Intervention				
	Exercise sessions, mean (range)	13.1 (0–24)	0	13.1 (12.2 to 14.0)
Primary health care, visits				
	General practice	18.2 (1.3)	19.5 (1.7)	−1.36 (−5.46 to 2.74)
	Physiotherapist	0.3 (0.1)	0.5 (0.2)	−0.27 (−0.81 to 0.27)
	Medical specialist	0.9 (0.2)	1.0 (0.2)	−0.05 (−0.57 to 0.48)
	Chiropractor	0	0.4 (0.03)	−0.40 (−0.77 to −0.03)
	Other^a^	2.0 (0.1)	2.2 (0.2)	−0.28 (−0.72 to 0.16)
Subtotal		21.4 (1.3)	23.7 (1.7)	−2.36 (−6.61 to 1.90)
Secondary health care, visits				
	Outpatient	8.4 (0.9)	8.5 (1.0)	−0.13 (−2.80 to 2.55)
	Emergency	0.2 (0.1)	0.5 (0.1)	−0.24 (−0.52 to 0.03)
Subtotal		8.6 (0.9)	9.0 (1.1)	−0.37 (−3.16 to 2.43)
Secondary health care, inhospital days				
	Orthopeadic	3.6 (0.4)	4.6 (1.1)	−0.95 (−3.32 to 1.42)
	Surgery^b^	0.3 (0.1)	1.2 (0.7)	−0.91 (−2.28 to 0.45)
	Medicine^c^	0.7 (0.3)	0.8 (0.3)	−0.12 (−0.92 to 0.68)
	Oncology^d^	0	0.4 (0.3)	−0.37 (−0.95 to 0.22)
	Other	0.3 (0.2)	0.3 (0.3)	−0.01 (−0.72 to 0.70)
Subtotal		4.8 (0.7)	7.2 (1.8)	−2.36 (−6.11 to 1.38)

Variables are expressed as the mean (bootstrap SE) number of outpatient visits or in-hospital days per patient during the 61 week follow-up period and the mean difference (95% confidence interval) between groups

^a^Other, includes visits at the dentistry, laboratory or foot care clinic

^b^Surgery, gastrointestinal, urology, plastic, thoracic

^c^Medicine, internal medicine, cardiology, medical gastroenterology, neurology, geriatrics, general practice

^d^Other, includes inhospital stay at oftamology, odontology or physiotherapy units

The average number of health care visits in primary and secondary care, including the number of inhospital days was not significantly different between groups, with the exception of visits with a chiropractor (Table 3). In total, 4 and 36 visits with chiropractor were registered in the intervention and control groups, respectively. 95% of the visits in the subgroup “other” had visited the dentist. None of the participants had visited psychologist funded by the national health care system during the follow-up period. Summarizing all inhospital days, 75% were to orthopaedic units.

### Cost

Participating in the supervised neuromuscular exercise program cost on average (SE) €326 (12.9) per patient (Table 4). A mean of 7.7 patients attended each session. Hence, the tariff to the physiotherapist was based on groups of eight equivalent to a cost of €186/session (https://fysio.dk/praksis/Overenskomst-og-takster/Almen-fysioterapi1/Almen-fysioterapi/). No differences between groups (€-132; 95% CI −3668 to 3405) were found for costs in primary or secondary health care sector (Table 4). The largest cost was, as expected, found for inpatient hospital stay.

**Table 4:** Costs of the intervention, outpatient and emergency visits and inpatient hospital stay during 61 weeks

		Intervention group (*n* = 84)	Control group (*n* = 81)	
				Mean difference (95% CI)
Intervention				
	Physiotherapy	326 (13)	0	326 (301 to 351)
Primary health care sector				
	General Practice	331 (28)	345 (29)	−14 (−92 to 64)
	Physiotherapist	22 (10)	98 (61)	−76 (−197 to 45)
	Specialists	99 (21)	97 (29)	2 (−68 to 72)
	Chiropractor	2 (1)	6 (2)	−4 (−9 to 0)
	Other^a^	75 (7)	83 (8)	−7 (−28 to 14)
Subtotal		530 (35)	629 (74)	−99 (−258 to 60)
Secondary health care sector				
	Outpatient	2240 (560)	1917 (317)	323 (−933 to 1579)
	Emergency	12 (4)	32 (11)	−20 (−43 to 4)
Subtotal		2252 (557)	1949 (316)	303 (−949 to 1555)
Secondary health care sector				
	Orthopaedics	11760 (572)	11695 (1038)	66 (−2250 to 2382)
	Surgery^b^	280 (180)	773 (363)	−493 (−1285 to 300)
	Medicine^c^	883 (322)	865 (350)	18 (−919 to 955)
	Oncology	0	213 (170)	−213 (−547 to 120)
	Other^d^	150 (149)	189 (169)	−40 (−494 to 415)
Subtotal		13074 (706)	13735 (1249)	−662 (−3478 to 2154)
TOTAL		16181 (1174)	16313 (1374)	−132 (−3668 to 3405)

Variables are expressed as the mean (bootstrap SE) per patient during the 61 week follow-up period and the mean difference (95% confidence interval) between groups. The monetary units are presented in EUR2012

^a^Other, includes visits at the dentistry, laboratory or foot care clinic

^b^Surgery, gastrointestinal, urology, plastic, thoracic

^c^Medicine, internal medicine, cardiology, medical gastroenterology, neurology, geriatrics, general practice

^d^Other, includes inhospital stay at oftamology, odontology or physiotherapy units

Patients attending exercise travelled on average 21.5 km (range 0.5–78.3 km) to the exercise facility and had a mean (SE) transportation cost of €137 (12.2). The mean (SE) informal time valuation was €302 (15.3) for the intervention period. When including patients’ expenses, the cost for the intervention increased to a mean (SE) of €765 (33.5) per patient during the intervention period.

### Cost-utility

**Fig. 2:**
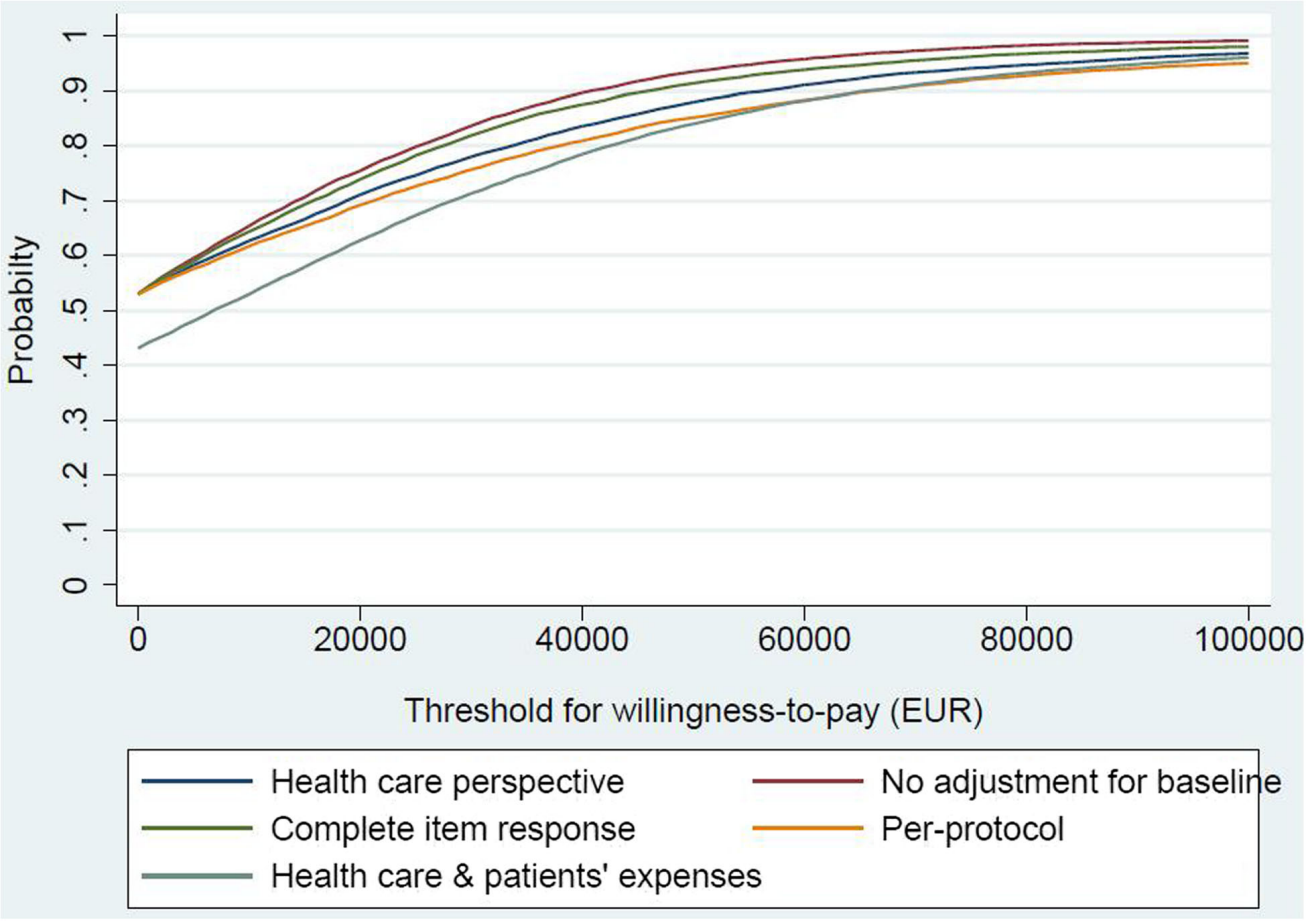
Cost effectiveness acceptability curves for incremental net monetary benefit to estimate the probability for the intervention being cost effective at conventional thresholds for willingness to pay. Health care perspective, Health Care Sector perspective (base-case analysis); Complete item response, only complete item response of the EQ-5D-3L included in the analysis; Health care & patients’ expenses, Health Care Sector and patients’ own expenses perspective; No adjustment for baseline, adjustments for baseline EQ-5D-3L scores were not included; Per-protocol, only patients attending 12 or more exercise sessions were included in the analysis

At conventional thresholds, decision-makers willingness to pay around €40,000 the probability for the intervention being cost-utile was estimated at 84%. Sensitivity analyses showed that the cost-utility result was robust (Fig. 2).

## Discussion

The present analysis demonstrates that a pre-operative 8-week supervised exercise intervention was cost-effective, showing a probability of 84% for decision-makers willingness to pay around €40,000 for a QALY gained. This is comparable to the typical threshold between £20,000 and £30,000 per QALY gained used by the British National Health Service when instituting new treatments.[34] Compared to care as usual, we found no overall additional health care cost during the first post-operative year despite adding 8 weeks of physiotherapist-supervised exercise prior to TKR and THR surgery. In return one can expect QALY gain over the following year.

This study is one of few RCTs that estimates costs and cost-effectiveness of exercise as treatment in patients with hip and knee OA, and, to our knowledge, the first to analyse cost-utility of exercise prior to THR and TKR. Previous cost-effectiveness analyses evaluating exercise, as the only intervention and not prior to surgery, in patients with knee OA found better health outcomes at lower costs, i.e. exercise was cost saving [13]. One RCT found that water-based exercise saved £123-175 per patient per year despite a relatively high intervention cost (£830 per patient) [35]. A second RCT found that both aerobic and resistance training were cost saving, $114 and $117, respectively, along with improvements in self-reported functioning [36]. A third RCT found QALY gains of 0.023 (SE 0.04) from class-based exercise compared to home-based exercise and a probability of 70% of being cost-effective at a willingness to pay of £30.000 [37]. Further, two RCTs have shown less total costs for patients with hip OA and chronic knee pain, respectively, following patient education and exercise programs compared to usual care [38, 39]. Comparing our study of exercise prior to OA surgery to studies evaluating exercise interventions in other patient groups, the results are quite similar. A Cochrane review of exercise for patients with heart failure reported a QALY gain of 0.03 and a probability of 90% for willingness to pay around 50.000 USD [40], group-based exercise for the prevention of falling showed an incremental cost per QALY gain of 72.700 AUD [41], and pelvic-floor muscle training showed cost-effectiveness with a probability of >70% for the willingness to pay around ₤50.000 [42].

We found that patients allocated to exercise had a lower total length of hospital stay and total cost during the follow-up period. The results were not statistically significant. However, should these findings not be due to random variation, a reduction of 2.4 days in hospital per patient and year is a relevant difference for the health care system. In 2012, a total of 8787 and 8008 patients underwent THR and TKR, respectively, at Danish hospitals [43, 44]. Extrapolating the resource use to the Danish THR and TKR population in 2012, exercise prior to surgery could potentially save 39.500 days in hospital per year. Seventy-five percent of all in hospital days during the 61 week period were at orthopaedic units, leaving 25% at other units. We did not ask the patients about comorbidities, but the data show that at least some had concurrent diseases. The second largest cost, after admission to orthopaedic units, was admission to internal medicine and cardiology units (Table 4). Over half of the population with hip and knee OA have been found to have concomitant cardiovascular disease and 86% of patients going through THR or TKR have one or more comorbidities [45, 46]. There have also been found significant associations between number and type of comorbidity and lower ADL, pain and HRQoL scores, with largest impact on ADL in THR patients, suggesting that functional limitations due to other diseases have to be taken into account to optimize outcome after THR and TKR [45, 46]. A national database study from Taiwan found a reverse dose-relationship of having coronary artery disease and dyslipidemia in patients attending physiotherapy due to symptoms from their OA, i.e. patients receiving a higher dose of physiotherapy showed a lowered risk of coronary artery disease and dyslipidemia [47]. Since comorbidities are common in OA and possibly can be influenced by the intervention given in our study, we find the inclusion of the total cost and resource use important for this study (Table 3 and 4).

The sensitivity analyses showed that the health care sector perspective using LTAP imputed data was robust as compared to complete item response, unadjusted and per-protocol analyses. The analysis using the health care sector plus patients’ expenses differed slightly from the base-case analysis showing a slightly lower probability for willingness to pay thresholds. This was expected since adding patients’ average expenses by €439 to the average cost of the intervention (€326) would result in higher costs for the intervention group, i.e. €765 versus €326 for the intervention per patient calculated with or without patient expenses, respectively. This study had a follow-up period of 61 week. We did not find it necessary to discount for costs or consequences as the study only passed one year by 9 weeks. Implementation cost of the intervention was not included in the analysis as the intervention today is available at 377 private and public physiotherapy clinics nationwide in Denmark [48]. Hence, the exercise program is already on the market in Denmark and could be implemented as standard care for patients undergoing THR or TKR without extra costs for education and training of care providers.

With regards to generalisability of the cost-utility results there are challenges in interpretation due to the vast differences in health care structure worldwide. In Denmark, all health care utilization and cost for different services and procedures performed in primary and secondary care are registered in national registers [22, 23]. To enable comparison to other health care structures, the overall average unit costs in this study can be found by dividing the cost estimates in table 4 with the resource use in table 3 or by viewing the tariff catalogues [49]. However, the efficacy of the intervention is well documented and it is of general interest to optimise the pre- as well as postoperative period with increased activities of daily living and through this a possible reduction in postoperative complications (e.g. joint stiffness, thrombosis/emboli) and a faster return to work for the younger part of this patient group.

### Strength and limitations

Some strengths of this RCT were a rigorous design by applying the CONSORT recommendations [30], evaluating a time horizon of one year, in which changes in ADL and HRQoL are expected to appear [50, 51], and using a common generic HRQoL measurement (EQ-5D-3L) to calculate QALYs [52]. Our sample size of 165 allowed us however to detect moderate, as opposed to small, effect sizes. We found a significant effect size of 0.59 favouring the exercise group in HOOS/KOOS quality of life, but the effect size of 0.39 in HOOS/KOOS sport and recreation function remained non-significant (Table 2). One limitation of this RCT was that the Danish national registers do not include costs for care delivered directly by the municipality. In Denmark, the municipality is responsible for post-operative care after hospital discharge, e.g. standard post-operative exercise and home-care. Although group allocation was stratified on municipality, differences in resource use and costs between the groups during the follow-up period may exist. Another limitation was that we had no data on patients’ work status (sick-leave, disability pension, retired or in the workforce) or OA-related medicine. The majority of participants in this study were assumed to be retired, as the retirement age in Denmark is 65 years and the average age in the study was 67.5 years at baseline. Even though the majority was assumed to be retired, a recent study has shown that the year after THR and TKR patients cost €6000 more compared to a reference population due to loss of employment income, use of medication and need for home care [53]. A broader societal perspective including also work status, medication and municipality-based services would therefore have been optimal. If this intervention would be implemented in routine clinical practice, the intervention could be provided in the primary care setting usually located within 15 km from peoples’ homes. The private pocket cost of transportation would then be reduced whereas program administration costs might increase and gains from economies of scale could be lost. This should be considered and balanced with the potential benefit of extra participation and/or compliance by patients sensitive to provider and/or transportation distance. Finally, of those meeting eligibility criteria for this study only 30% were included which may impact external validity.

## Conclusion

Preoperative supervised neuromuscular exercise for 8 weeks was found to be cost-effective in patients scheduled for THR and TKR surgery at conventional thresholds for willingness to pay. One-year clinical effects were small to moderate and favoured the intervention group, but only statistically significantly so for quality of life measures.

## Abbreviations

ADL: Activities of Daily Living
CEAC: Cost-Effectiveness Acceptability Curves
CI: Confidence Interval
CONSORT: Consolidated Standards of Reporting Trials
DKK: Danish Krona
EQ-5D-3L: European Quality of Life 5-Dimension 3-Level Health Outcome
HOOS: Hip disability and Osteoarthritis Outcome Score
HRQoL: Health Related Quality of Life
KOOS: Knee injury and Osteoarthritis Outcome Score
LOCF: Last Observation Carried Forward
LTAP: Linear Trend At Point
OA: Osteoarthritis
QALY: Quality Adjusted Life Year
RCT: Randomised Controlled Trial
SD: Standard Deviation
SE: Standard Error
THR: Total Hip Replacement
TKR: Total Knee Replacement
USD: US Dollar
